# PHD2 in tumour angiogenesis

**DOI:** 10.1038/sj.bjc.6605682

**Published:** 2010-05-11

**Authors:** D A Chan, A J Giaccia

**Affiliations:** 1 Department of Radiation Oncology, University of California, 2340 Sutter Street, S-332, Box 1331, San Francisco, CA 94143-1331, USA; 2 Department of Radiation Oncology, Stanford University School of Medicine, 269 Campus Drive, CCSR-South, Room 1255, Stanford, CA, 94305, USA

**Keywords:** PHD2, tumour angiogenesis, HIF

## Abstract

Originally identified as the enzymes responsible for catalysing the oxidation of specific, conserved proline residues within hypoxia-inducible factor-1*α* (HIF-1*α*), the additional roles for the *p*rolyl *h*ydroxylase *d*omain (PHD) proteins have remained elusive. Of the four identified PHD enzymes, PHD2 is considered to be the key oxygen sensor, as knockdown of PHD2 results in elevated HIF protein. Several recent studies have highlighted the importance of PHD2 in tumourigenesis. However, there is conflicting evidence as to the exact role of PHD2 in tumour angiogenesis. The divergence seems to be because of the contribution of stromal-derived PHD2, and in particular the involvement of endothelial cells, *vs* tumour-derived PHD2. This review summarises our current understanding of PHD2 and tumour angiogenesis, focusing on the influences of PHD2 on vascular normalisation and neovascularisation.

The rapid growth of tumours results in their surpassing the capacity of the established circulatory system to supply the developing tumour with oxygen and nutrients as well as remove waste products. For a tumour to expand, it must respond to these assaults. Thus, a necessary step in the progression of any solid tumour is adaptation to low oxygen or hypoxic conditions. Tumour cells can respond to hypoxia by increasing oxygen delivery or acclimating to decreased oxygen availability. The hypoxia-inducible factor (HIF) family of transcription factors mediates both the systemic and cellular response to hypoxia.

As master regulators of oxygen homeostasis, HIF-1 and HIF-2 must be tightly controlled to prevent the inappropriate expression of hypoxia-induced genes. HIF-1 and HIF-2 are heterodimeric transcription factors that are regulated at the post-translational level. They are composed of a constitutive *β*-subunit (also known as ARNT) and an oxygen-sensitive *α*-subunit. The *α*-subunit is hydroxylated on two conserved proline residues by a family of prolyl hydroxylases (Ivan *et al*, 2001; Jaakkola *et al*, 2001; Yu *et al*, 2001). The hydroxylation of the *α*-subunits allows the von Hippel-Lindau (VHL) E3 ubiquitin ligase to interact, adding ubiquitin ladders and targeting the *α*-subunit to the proteasome for degradation (Maxwell *et al*, 1999). HIF transactivation is also regulated by a third hydroxylation. This post-translational modification of a conserved asparagine residue (Asp 803 of HIF-1*α* and Asp 831 of HIF-2*α*) prevents the interaction of HIF with p300, a transcriptional coactivator (Mahon *et al*, 2001; Lando *et al*, 2002a, ). Hydroxylation of the *α*-subunits of HIF-1 and HIF-2 have critical roles in both the stability and activity of HIF.

## Hypoxia-inducible factors and cancer

Under pathological conditions, strikingly in cancer, HIF becomes deregulated and has a key role in the development of tumours. Clinically, HIF is overexpressed in a variety of cancers, predicting poorer prognosis (Zhong *et al*, 1999; Semenza, 2002). Experimentally, HIF is required for the growth of solid tumours (Ryan *et al*, 1998, 2000). Deletion of *Hif-1α* in teratoma tumour models resulted in smaller tumours compared with teratomas containing wild-type Hif-1*α*. Using transgenic mice, Hif-1*α* has been shown to be an important factor in the development and metastatic spread of a variety of cancers, including those of the breast and brain (Tang *et al*, 2004; Liao *et al*, 2007; Du *et al*, 2008). Conversely, in embryonic stem cells, deletion of Hif-1 reduced hypoxia-induced apoptosis while increasing proliferation, resulting in larger tumours (Carmeliet *et al*, 1998). The role of HIF in tumourigenesis is further complicated by disparate findings for HIF-1 and HIF-2 (Acker *et al*, 2005; Gordan *et al*, 2008). However, tumour promoting or activating mutations in the HIF sequence have not been identified. A key upstream regulator of HIF is the VHL E3 ubiquitin ligase. VHL is a well-known tumour suppressor gene and VHL disease is characterised by a distinct subset of highly vascular tumours, notably renal carcinoma, angiomas, and hemangioblastomas (Kaelin, 2007). Mutation or silencing of *VHL* accounts for >80% of all renal carcinomas, the most common form of kidney cancer (Gnarra *et al*, 1993, 1994; Nickerson *et al*, 2008). Nevertheless, inactivation of VHL by itself cannot explain the broader variety of tumour types that have elevated HIF protein.

**Figure 1:**
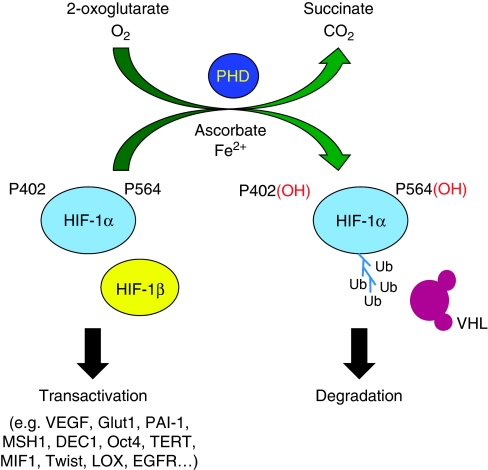
In the presence of oxygen, a family of prolyl hydroxylases oxidises HIF-1*α*, leading to interaction with VHL and subsequent degradation. Conversely, when the prolyl hydroxylases are not active, HIF-1*α* is stabilised, interacts with HIF-1*β*, and is transcriptionally active.

Another key regulator of HIF is the family of prolyl hydroxylases (PHD1, PHD2, and PHD3) that modify HIF to be recognised by VHL. The prolyl hydroxylases require oxygen and 2-oxyglutarate as substrates and Fe(II) and ascorbate as cofactors, resulting in oxidation of two highly conserved proline residues (Figure 1). Recently, a paper by Ladroue *et al* (2008) identified a patient who had a single amino acid substitution in the iron-binding pocket of PHD2. The patient presented with congenital erythrocytosis and multiple paragangliomas. Furthermore, Kato *et al* (2006) reported a high mutation rate of PHD2 in endometrial cancers. Mutations in succinate dehydrogenase and fumarate hydratase, both components of the citric acid cycle, have also been linked to tumour-prone syndromes, resulting in part because of inhibition of the PHDs (Isaacs *et al*, 2005; Ricketts *et al*, 2009). Overexpression of PHD1 has been shown to inhibit tumour growth (Erez *et al*, 2003), whereas PHD3 is downregulated in colorectal cancers (Xue *et al*, 2009). The complexity of interactions between HIFs and the PHDs were highlighted by Henze *et al* (2010) who identified a negative feedback loop in gliomas. Taken together, these findings suggest that other negative regulators of HIF, such as the prolyl hydroxylases, may contribute to tumourigenesis. However, the role of PHDs in tumourigenesis is poorly defined.

## The role of PHD2 in tumour angiogenesis

Of the identified HIF prolyl hydroxylases, PHD2 is thought to be the key oxygen sensor regulating HIF (Berra *et al*, 2003). Silencing *PHD2* through RNA interference increased HIF-1*α* levels under normoxic conditions. This effect was not observed with either *PHD1* or *PHD3*. Furthermore, several groups independently generated genetic knockout mice of the different *Phd*s (Takeda *et al*, 2006; Aragones *et al*, 2008; Minamishima *et al*, 2008). Phd1 and Phd3 homozygous knockout mice appeared phenotypically normal with the expected Mendelian ratios (Takeda *et al*, 2006). Meanwhile, homozygous knockout of Phd2 was embryonic lethal between days 12.5 and 14.5, because of vascular defects of the placenta and the heart. Moreover, conditional knockout of Phd2 increased vascular and capillary density, vessel branching, and recruitment of vascular smooth muscle cells, whereas conditional knockout of Phd1 and Phd3 did not have these vascular effects (Takeda *et al*, 2007). Taken together, these data suggest that PHD2 may have an important role in regulating HIF and angiogenesis.

We recently identified PHD2 as a mediator of potent tumour angiogenesis pathways (Chan *et al*, 2009) whereas Mazzone *et al* (2009) reported a different function of PHD2 in vessel normalisation. We began our studies by analysing mRNA and protein expression levels of PHD2 in human tumours. In colorectal carcinomas, PHD2 levels of mRNA and protein were both decreased in the tumour compared with non-involved, adjacent normal colon tissue, suggesting that loss of PHD2 may influence the tumour development. To further investigate, we used shRNA to stably silence PHD2 in several different human cell lines, including three colorectal cell lines and a pancreatic cell line. In each of these lines, knocking down PHD2 levels did not affect *in vitro* cell growth. It should be noted that we did not challenge these cells to hypoxic stress or additional PHD inhibition. Henze *et al* (2010) found that inhibiting PHD activity by hypoxia or DMOG reduced glioma tumour cell survival *in vitro*. However, when we implanted our cell lines as xenografts into the flanks of immunocompromised mice, tumour growth was significantly and dramatically increased compared with wild-type control cells. As the best-characterised target of PHD2 is HIF, we then investigated whether the enhanced tumour growth of PHD2 loss is dependent on HIF

Using HCT116 cells, a colon carcinoma, deleted for HIF-1*α* (Dang *et al*, 2006), we then silenced PHD2 and implanted these cells as tumours. Tumours that lacked HIF and PHD2 grew faster than control tumours that only lacked HIF. This provocative finding suggests that PHD2 has additional functions that are independent of HIF.

To determine the mechanism of why tumours with PHD2 silenced grew faster, we sectioned the tumours. TUNEL analysis showed no difference between control and knockdown tumours, showing that the wild-type tumours were not smaller because of an increase in apoptosis. However, Ki67 expression, a marker of proliferation, indicated that the PHD2-silenced tumours grew better than wild-type tumours *in vivo*. These results suggested that PHD2 disruption in tumour cells altered their interaction with the tumour microenvironment, allowing for more efficient growth. We further stained the tumour sections for CD31, a marker of blood vessels, which showed a three- to four-fold increase in tumour blood vessels in the tumours with PHD2 silenced. Similarly, several additional groups have also found that PHD2 can influence tumour growth through its effect on angiogenesis. Lee *et al* (2008) showed that a reduction in PHD2 leads to enhanced tumour growth and enhanced tumourigenesis. In reciprocal experiments, Matsumoto *et al* (2006, 2009) found that 2-oxogluturate, a substrate of PHD2, reduced both tumour growth and angiogenesis. Examining the effect of Phd2 deletion on endothelial cells directly, Takeda and Fong (2007) found that loss of Phd2 impaired proliferation. Thus, it seemed likely that the enhanced tumour blood supply of the PHD2 knockdown tumour was providing the necessary components to increase tumour growth.

We then investigated whether the PHD2 knockdown cells were secreting factors capable of influencing angiogenesis. Using a standard *in vitro* angiogenesis assay of endothelial cell tube formation, conditioned media from PHD2 knockdown HCT116 cells were able to cause primary endothelial cells plating on matrigel to aggregate, forming complex, tube-like structures. Conditioned media from control HCT116 cells, which do not have PHD2 knocked down, however, lacked the components to induce the endothelial cells to branch. Subjecting the conditioned media to an angiogenesis antibody array, we revealed that PHD2 consistently regulates angiogenin (ANG) and IL-8, two known soluble pro-angiogenic factors. Conditioned media from HCT116 cells that had PHD2 knocked down had elevated protein levels of ANG and IL-8. Notably, the levels of VEGF were unchanged by PHD2 levels. Silencing either ANG or IL-8 impaired both angiogenesis and tumourigenesis. These results suggested that PHD2 normally functions to inhibit angiogenesis and that silencing PHD2 promotes angiogenesis.

Tumour vasculature is actually regulated by two complementary processes: angiogenesis, which is local sprouting, and vasculogenesis, which is the *de novo* production of new blood vessels. Angiogenesis is the formation of new blood vessels from pre-existing vessels. This local angiogenesis can also send signals to the bone marrow, which can in turn release precursor or progenitor cells to induce neovascularisation. This process of blood vessel formation by *de novo* production of endothelial cells, or vasculogenesis, requires tumour cells to interact with stromal cells and circulating bone marrow-derived cells (BMDCs). We then investigated whether PHD2 knockdown affected recruitment of BMDCs to the growing tumour vasculature. To determine this we stained for two BMDC markers, CD11b and CD45. CD11b is a marker of myeloid–monocytic precursors, whereas CD45 is a myeloid marker. PHD2 silencing increased mobilisation of BMDCs to the growing tumour, whereas silencing of ANG and IL8 impaired this mobilisation. Thus, PHD2 functions to regulate both angiogenesis and vasculogenesis through ANG and IL-8.

The PHD2 regulation of tumour vasculature through ANG and IL-8 is independent of HIF. Cummins *et al* (2006) showed that PHD1 could regulate IKK*β*, an inhibitor of NF-*κ*B, which suggested that PHD2 might also regulate NF-*κ*B. In PHD2-silenced cells, NF-*κ*B activity was elevated and mutation of the NF-*κ*B sites in the promoters of ANG and IL-8 impaired NF-*κ*B activation. Furthermore, IL-8 is a well-characterised NF-*κ*B target and NF-*κ*B bound to the promoter of ANG, as determined by chromatin immunoprecipitation, showing that the influence of PHD2 on the tumour vasculature is mediated through NF-*κ*B activity on ANG and IL-8, two known pro-angiogenic factors. Transient silencing of p65, an essential subunit of the NF-*κ*B complex, impaired *in vitro* angiogenesis. Using breast cancer data sets, there was a strong, inverse correlation between PHD2 mRNA levels and NF-*κ*B activity profiles as well as negative relationship between PHD2 and CD31 mRNA levels, as determined by microarray analysis. Furthermore, NF-*κ*B activity profiles positively correlated with an increase in CD31 mRNA. Taken together, these data illustrate that PHD2 can regulate the tumour vasculature through an HIF-independent mechanism but relies in part on inhibition of NF-*κ*B and the downstream targets of ANG and IL-8.

## Stromal PHD2 contributions to metastasis and vessel normalisation

In a parallel study, Mazzone *et al* (2009) investigated the stromal role of PHD2 in the development of tumours. Interestingly, using genetically modified mice that were heterozygous for *Phd2*, they saw no difference in ectopic, primary tumour growth. In an orthotopic, pancreatic model, tumours grown in wild-type mice were more invasive and had more metastatic disease compared with *Phd2*^+/−^ mice. This phenotype was attributed to higher intravasation of tumour cells into blood vessels of wild-type *vs Phd2* heterozygous animals. The *Phd2* heterozygous animals had higher expression of VE-cadherin and less hypoxia based on pimonodozole staining, oxymetry, and HIF protein levels. Tumours implanted into *Phd2*^+/−^ mice also had less necrosis and lower metabolic rates. The same researchers showed that vessels of tumours implanted in heterozygous mice were normalised compared with those of wild-type mice. The vessel normalisation of tumours in *Phd2* heterozygous mice resulted in less intravasation of tumour cells and consequently less metastasis.

In contrast to our findings, this group (Mazzone *et al*, 2009) saw equivalent tumour vessel density between the heterozygous *Phd2* and wild-type mice. However, despite equal numbers of tumour vessels, the tumours of the *Phd2*^+/−^ mice had smoother, more regular, and more mature blood vessels that were not as malformed or as leaky as tumours implanted in wild-type mice. These researchers suggested that the tumour vessels formed in the *Phd2* heterozygous mice were ‘normalised,’ comparable to normal blood vessels. Examining the angiogenic profile of *Phd2* heterozygous endothelial cells as well as tumour endothelial cells from tumours implanted into *Phd2*^+/−^ mice, they found higher soluble Flt1 and VE-cadherin expression at the mRNA and protein levels, respectively. Soluble Flt1 and VE-cadherin are two angiogenic factors implicated in vessel normalisation and more specifically, cell motility and cytoskeletal reorganisation. Interestingly, in response to VEGF, endothelial cells heterozygous for *Phd2* proliferated slower, had decreased motility, less lamellipodia formation, and less apoptosis. The effects of sFlt1 and VE-cadherin were dependent on HIF2 and not HIF1, as transient silencing of only HIF2 decreased levels of both angiogenic factors. Taken together, Mazzone *et al* (2009) found that stromal PHD2 functions to regulate vessel normalisation, providing tighter junctions and preventing metastatic spread.

## Conclusions: vessel normalisation *vs* increased vasculature

**Figure 2:**
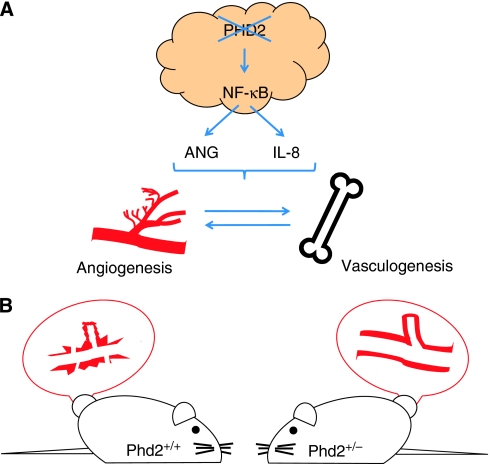
(**A**) Loss of PHD2 in a tumour results in activation of NF-*κ*B. In turn, NF-*κ*B upregulates ANG and IL-8 that promote both angiogenesis and bone marrow-derived cell recruitment. These two complementary pathways drive tumour growth. (**B**) Tumours implanted into Phd2^+/+^ mice have irregular and leaky vessels, whereas those implanted into Phd2^+/−^ mice have ‘normalised’ vessels.

The simplest explanation for these apparent discrepancies between our study and that of Mazzone *et al* (2009) is differences in the experimental models. We examined the influence of PHD2 loss on tumour growth, whereas they examined the influence of the host on tumour growth. We used shRNA to silence PHD2 in human cell lines, which were then implanted as xenografts into immunocompromised mice. In comparison, Carmeliet *et al* (1998) used syngeneic mouse cell lines inoculated into the flanks or orthotopically implanted into immunocompetent but heterozygous *Phd2* mice. Our study examined the role of PHD2 in a tumour to contribute to its growth through angiogenesis and recruitment of bone marrow-derived cells (Figure 2A). The complimentary study examined the function of stromal PHD2 in regulating tumour metastasis (Figure 2B). Simultaneous investigations into the participation of both tumour and host will be necessary to further delineate the responsibilities of PHD2. Furthermore, using spontaneous tumour models as well as specific knockout in various cell types may help elucidate HIF-dependent and HIF-independent functions of PHD2.
